# Hydroxyapatite ocular implant and non-integrated implants in eviscerated patients

**Published:** 2015

**Authors:** S Gradinaru, V Popescu, C Leasu, S Pricopie, S Yasin, R Ciuluvica, E Ungureanu

**Affiliations:** * Ophthalmology Department, “Carol Davila” University of Medicine and Pharmacy, Bucharest, Romania; ** Anatomy Department, “Carol Davila” University of Medicine and Pharmacy, Bucharest, Romania; *** University Emergency Hospital Bucharest, Bucharest, Romania

**Keywords:** hydroxyapatite, ocular implant, ocular prosthesis

## Abstract

**Introduction:** This study compares the outcomes and complications of hydroxyapatite ocular implant and non-integrated ocular implants following evisceration.

**Materials and Methods:** This is a retrospective study of 90 patients who underwent evisceration for different ocular affections, in the Ophthalmology Department of the University Emergency Hospital Bucharest, between January 2009 and December 2013. The outcomes measured were conjunctival dehiscence, socket infection, implant exposure and extrusion rate.

**Results:** Forty-three patients had the hydroxyapatite implant (coralline–Integrated Ocular Implants, USA or synthetic–FCI, France) and forty-seven received non-integrated ocular implants (24 acrylic and 23 silicone). Five cases of socket infection, thirteen cases of extrusion and two cases of conjunctival dehiscence were encountered.

**Conclusions:** There was a higher rate of conjunctival dehiscence with hydroxyapatite ocular implant, but implant extrusion and socket infection were found in non-integrated ocular implants.

## Introduction

From the time of the first integrated ocular implants, the introduction following the initial clinical work, by Perry A [**1**], hydroxyapatite was considered a real biomaterial for medicine.

Following the removal of the content of the eye, frequently after infections, trauma or end stage neovascular glaucoma, the content of the scleral sac was to be filled with an implant to replace the volume in the orbit (bony cavity surrounding the eye) that was occupied by the eye, an ocular implant that maintained the natural aspect of the orbit and provided support for the artificial eye (ocular prosthesis).

Evisceration – is a surgical procedure in which the content of the eye is removed (uvea, retina, lens, aqueous humor, vitreous humor) by preserving the sclera, Tenon capsule, conjunctiva and optic nerve. In most of the cases, the cornea is also removed, but it can be preserved for the enhancement of the ocular volume, especially for microphthalmia and nanophthalmia.

The indications for evisceration are the following:

• Painful blind eye [**2**], without light perception (especially in neovascular glaucoma, infectious endophthalmitis [**3**,**4**], etc.);

• Cosmetic improvement for disorganized eye;

• Extended ocular trauma recent than 24 hours;

• Phthisis bulbi and severe microphthalmia.

Calcium phosphates are the most abundant inorganic constituents of the living beings’ hard tissue, which provide bone and teeth with hardness, density and mechanical stability.

Hydroxyapatite (Ca10(PO4)6(OH)2) is the main calcium phosphate in these tissues and the crystal unit cell comprises two entities.

**Table 1:** Chemical composition of hydroxyapatite

Calcium	38.89% Ca	55.82% CaO
Phosphorus	18.50% P	42.39% P2O5
Hydrogen	0.20% H	1.79% H2 O
Oxygen	41.41 % O	

Through ages, various materials were used as ocular implant (wood, gold, cork, etc.) and hydroxyapatite history dates back as far as 1885, in the manuscripts by Mules.

**Fig. 1:**
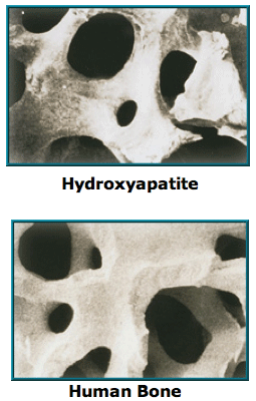
Microscopic aspect of hydroxyapatite porous structure and human bone (reproduced with permission from http://ioi.com)

The first ocular implant made of hydroxyapatite was implanted in 1985, by Dr. Arthur Perry, and was made from a specific genus of reef-building coral, which was changed from calcium carbonate to calcium phosphate, by hydrothermal exchange reaction, which also removed proteins and residua. Because this is an inert porous substance, with the same regular system of interconnecting pores that resembles the Havesian system of human bone (**Fig. 1**) it is not treated as a foreign substance, by the body, in contrast to most other implants used in the past, and, its porous architecture provides a framework for fibrovascular ingrowth. As the implant vascularizes, it becomes incorporated into the ocular tissue, which helps reduce the chance of implant migration, a problem not uncommonly seen with previous implants [**5**,**6**].

Coralline hydroxyapatite has realized the base for synthetic hydroxyapatite and there are many synthetic routes and preparation methods for hydroxyapatite: wet synthesis, solid-state reaction, sol-gel process, sonochemical synthesis and the hydrothermal route. Synthesis in solution implies foreign ions, since most calcium salts are sparingly soluble and only few like nitrates, chlorides or acetates are suitable for this method. Solid state routes rely on calcium phosphates and very prolonged heating, milling, palletizing and reheating cycles in order to achieve homogeneity and complete reaction [**7**]. The sol-gel method has some advantages such as particle size and coating forming, but is not a cheap method. Hydrothermal conditions are limited because of limited amount obtained.

The first-generation of synthetic hydroxyapatite was not 100% hydroxyapatite as the coralline hydroxyapatite, because it contained 3.2% calcium oxide, the implant was heavier and less porous than the original hydroxyapatite implant. The second-generation implant was more porous than the first and was chemically identical to coralline hydroxyapatite, but was very fragile and crumbled easily. The third-generation implant was more porous than its predecessors, and was not as fragile as was the second-generation implant [**8**].

**Fig. 2:**
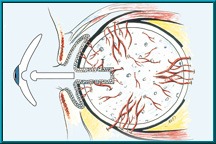
Peg implantation after adequate vascular ingrowth from the orbit (reproduced with permission from http://ioi.com)

Technical procedure: The ocular implant was surgically placed within the orbit at the time the eye was removed, with the patient under general anesthesia and the tissues (sclera and/ or conjunctiva and cornea) closed over the implant. The risks were kept to an absolute minimum, e.g. the risk of postoperative infection was minimized by a strict aseptic surgical technique and by the use of intraoperative and postoperative antibiotics. A temporary conformer (a clear plastic spacer) was then placed on top of the tissue covering the implant and under the eyelids to maintain the space for the future ocular prosthesis. In order to enhance the movement of the eye, the ocular prosthesis was attached to the implant by means of a peg. The peg’s biomaterial was made of titanium and it was introduced in the implant trough a hole drilled in the implant on a second surgical procedure. The peg placement procedure could only be performed after the implant had an adequate vascular ingrowth from the orbit, usually about six months after implantation (**Fig. 2**). This second procedure may also require general anesthesia but it can be performed under local anesthetic with/ without sedation [**9**].

The hydroxyapatite implant provides a series of advantages:

• The implant is easily accepted by the organism due to the similarity in structure with the other human tissues (bone, dental material);

• The risk of migration of the implant is reduced due to vascular ingrowth;

• Introducing the Peg permits a better movement of the ocular prosthesis.

The implant has a number of disadvantages: the costs of the implant and the second stage drilling procedure.

## Materials and methods

The aim of the study was to evaluate the efficacy and complications of the hydroxyapatite ocular implant compared to the non-integrated ocular implants in the eviscerated patients, in the University Emergency Hospital Bucharest, from January 2009 to December 2013. All the patients had an informed consent signed for the surgical procedure and an informed consent for the participation in this clinical study. Three surgeons from the Ophthalmology Department performed the evisceration surgeries, followed by the implantation of non-integrated ocular implant and one surgeon performed the evisceration surgery, followed by the implantation of a hydroxyapatite ocular implant.

The patients’ case reports and intraoperative reports were retrieved to record the type of implant, implant and socket complications. Implant complications included extrusion, conjunctival dehiscence and implant exposure. Socket complication was considered an ocular infection.

In this study, only patients who underwent the same standard surgical procedure for evisceration and ocular implantation were considered. The procedure for non-integrated ocular implant was the same for all the surgeons, consisting of 360-degree corneal limbus peritomy. The extraction of the uvea, retina, lens and vitreous was performed by using a curette and the cauterization of ophthalmic artery was also performed. The non-integrated ocular implant (acrylic or silicone) was placed, followed by the closure of the scleral sac, Tenon’s and conjunctiva.

The procedure for hydroxyapatite ocular implant was the same in all the patients and consisted of the same steps for the evisceration. The hydroxyapatite implant’s size was the following in all cases: 16 mm spheres (from FCI –France and Integrated Ocular Implants-USA) that were placed in the scleral sac were collected, and 4 posterior sclerectomies were performed in order to obtain fibrovascular ingrowth. The scleral sac, Tenon’s and conjunctiva were sutured.

All the patients had conformers placed; antibiotics and anti-inflammatory drugs were given for 5 days. After 6 weeks, the patients were referred for prosthesis fitting.

## Results

A total of 90 patients were enucleated, age ranging from 24 to 79 years. The main indication for evisceration was painful blind eye, and the second indication was trauma. All the patients received implants at the moment of surgery as first choice. Forty-three patients received hydroxyapatite integrated ocular implant and forty-seven received non-integrated ocular implants (24 silicone and 23 acrylic). There were five cases of socket infection in our series: four cases with non-integrated ocular implants and one with a synthetic hydroxyapatite (FCI-France). All five cases were followed by implant extrusion. There were another eight cases of implant extrusion with non-integrated ocular implants following the socket contracture.

There were 2 cases of scleral sac and conjunctival dehiscence followed by implant exposure in the hydroxyapatite ocular implant group, all the cases underwent surgery with oral mucosa grafting but one required placenta membrane tissue graft (multiple grafting).

## Discussion

From the biocompatibility point of view, all the materials offered excellent scaffold for ocular implantation and rehabilitation of an already suffering patient, yet the hydroxyapatite ocular implant (coralline or synthetic) possessed a series of advantages that included lower rates of extrusion, migration and resistance to infections. Shields et al [**10**] had a large database without implant extrusion, but with conjunctival dehiscence and implant exposure. Although the surgical technique was one of the factors that were responsible for the conjunctival dehiscence, in our study, only one surgeon performed the evisceration surgery with hydroxyapatite ocular implant, so, the technique was standardized in this study. Nunery et al [**11**] suggested that conjunctival dehiscence could be avoided by deep implantation.

## Conclusions

In our series, the implant complications and socket complications occurred mainly in the non-integrated ocular implants, compared to hydroxyapatite ocular implant.

Through years, hydroxyapatite ocular implant has gained popularity, but the cost is much higher than an acrylic or silicone sphere, so it is important to note that, for a vast majority of patients, it is not affordable with respect to a higher rate of complications for the acrylic or silicone spheres. In addition, there is a need for a better patient information and better case selection.

**Acknowledgments**

This paper is partially supported by UEFISCDI, financed from the European Social Fund and by the Romanian Government under the contract number PN-II-PT-PCCA-2013-4-0584.

**Sources of funding**

This paper is partially supported by UEFISCDI, financed from the European Social Fund and by the Romanian Government under the contract number PN-II-PT-PCCA-2013-4-0584.

**Disclosures**

None
